# Retraction: Hypothermia Protects the Brain from Transient Global Ischemia/Reperfusion by Attenuating Endoplasmic Reticulum Response-Induced Apoptosis through CHOP

**DOI:** 10.1371/journal.pone.0249055

**Published:** 2021-03-18

**Authors:** The PLOS ONE Editors

Following the publication of this article [1], concerns were raised regarding Figs 2, 3 and 4. Specifically,

- Panel A of Fig 2A appears similar to panel A of Fig 3A and panel A of Fig 4A.
- Panels B and C of Fig 3A appear similar to Panels B and C of Fig 4A, respectively.
- The GAPDH panels of Fig 2B appear similar to the GAPDH panels of Fig 3B.

The authors have indicated that the underlying data for this study cannot be shared due to data confidentiality. The unavailability of the original data underlying the published result is a breach of the PLOS data availability policy.

In light of the concerns affecting multiple figure panels that question the validity of these data and the unavailability of the raw data underlying the published results, the *PLOS ONE* Editors retract this article.

MW neither agreed nor disagreed with the retraction. XL, HC, YG, FM, FS, YB, and YL either could not be reached or did not respond directly.
